# Disruption to Substance and Opioid Use Disorder: The Deep South Substance and Opioid Use Rural Training Grant

**DOI:** 10.1093/geroni/igab046.2018

**Published:** 2021-12-17

**Authors:** Rebecca Allen, Lindsey Jacobs, Bruce Rybarczyk

## Abstract

The primary objective of this symposium is to describe our integrated, interprofessional behavioral health training program in substance use and opioid use disorders (SUD/OUD) across the adult lifespan (19 to 80) within our clinical psychology graduate program in the Deep South. Due to the COVID-19 pandemic, our assessment, treatment, and prevention delivery has occurred via telehealth. The first paper describes our Clinical Training Model in two federally qualified health centers (one peri-urban and one rural) and one residential drug and alcohol rehabilitation program. Graduate and undergraduate students provide prevention, assessment, and treatment with an emphasis on 1) mindfulness-based relapse prevention, 2) literacy-adapted treatment for chronic pain, and 3) trauma and recovery. The second paper describes the participant population. Specifically, participants (N = 105) receiving prevention, assessment and treatment services report high levels of substance and opioid use and are underserved, impoverished, and have low levels of education and health/mental health literacy. The third paper explores the relation of age, adverse childhood experiences, and PTSD symptoms within the context of substantial or severe SUD/OUD. The final paper describes issues surrounding telehealth delivery in the rural south with underserved populations. The discussant, an expert in integrated, interprofessional telehealth delivery across the adult lifespan, will provide insight on program sustainability and dissemination. Given the pronounced need for SUD/OUD treatment in underserved populations with attention to the intersection of age and urban/rural residence, this project is poised to make a substantive impact across the adult lifespan.

